# A Systematic Review of the Anti-Inflammatory and Immunomodulatory Properties of 16 Essential Oils of Herbs

**DOI:** 10.1155/2020/8878927

**Published:** 2020-12-07

**Authors:** Xu Zuo, Yinuo Gu, Chao Wang, Jinrong Zhang, Jing Zhang, Guoqiang Wang, Fang Wang

**Affiliations:** Department of Pathogeny Biology, College of Basic Medical Sciences, Jilin University, Changchun 130021, China

## Abstract

**Background:**

Inflammation is a host defense mechanism in the body after it is infected and damaged. If inflammation is not treated in time, then it may cause a variety of diseases, such as cancer and autoimmune diseases. Herbal essential oils are natural extracts that can suppress inflammation effectively and are expected to be used in therapeutic drugs for anti-inflammatory diseases in the future. *Aim of the review*. We review the anti-inflammatory and immunomodulatory effects of essential oils derived from 16 herbs. *Materials and methods*. We searched the literature of the fields of anti-inflammatory and immunomodulatory herbal essential oil activity published in English within the past five years via databases (PubMed, EMBASE, Scopus, and The Web of Science).

**Results:**

A total of 1932 papers were found by searching, and 132 papers were screened after removing duplicates and reading article titles. Fifteen articles met the requirements to be included in this review. Among those selected, 11 articles reported in vivo research results, and 10 articles showed research results.

**Conclusion:**

Essential oils extracted from herbs can reduce inflammation by regulating the release of inflammatory cytokines involved in multiple signalling pathways. Herbal essential oils are expected to be developed as anti-inflammatory drugs.

## 1. Introduction

Inflammation is a defense mechanism against infection and tissue damage [1]. Inflammation and proinflammatory cytokines, reactive oxygen species (ROS), lipid extraction medium: arachidonic acid (AA), hydrolases, transcription factors, etc., are closely related [2]. When not stopped in time, inflammation can cause diabetes and cancer [3], arthritis, Alzheimer's disease, atherosclerosis, cardiovascular disease, eye diseases, and autoimmune diseases, including inflammatory bowel disease [4]. Some clinical and physiopathological data also showed that inflammation could also affect children with inflammatory bowel disease, uveitis, and juvenile idiopathic arthritis by slowing their growth in height and weight [5]. Inflammation seriously affects people's lives.

Inflammation can usually be classified into two categories according to its course: acute inflammation and chronic inflammation. The immune system plays a key role in determining whether inflammation is acute or chronic. The activation of inflammation is closely related to immune cells and biological molecules. In particular, innate lymphoid cells (LCs), with multiple immune functions, play important roles in inflammatory diseases [6]. Infiltration of macrophages and neutrophils is a feature of acute inflammation, and infiltration of T lymphocytes and plasma cells is a feature of chronic inflammation [7]. These cells play important roles in the inflammatory response.

Steroidal and nonsteroidal anti-inflammatory agents are commonly used in the clinical treatment of inflammatory diseases. Long-term use of these drugs can cause serious adverse reactions, such as gastrointestinal tract, cardiovascular, and liver abnormalities [8]. Finding a safe and effective drug to control inflammation has always been a challenge. In recent years, Chinese herbal oils have been rated as the safest, most promising anti-inflammatory drug candidate [2].

Recently, it has been shown that a variety of active components of traditional Chinese medicine regulate the body's immune state to attenuate inflammation [9–11]. Volatile Chinese medicine oils are mixtures of multiple compounds [12]. The main components are monoterpene and sesquiterpene, among others, with anti-inflammatory, antibacterial, acaricidal, antiviral, sedative, antianxiety, and antidepression properties [13]. The anti-inflammatory effect of volatile oils of traditional Chinese medicine is mainly realized through the regulation of cyclooxygenase (COX) and induction of nitric oxide synthase (iNOS) and a variety of cytokines, which play roles in the inflammation process [14]. In recent years, essential oils of traditional Chinese medicine have been described as potential sources for the development of new drugs.

## 2. Materials and Methods

The review was conducted following the Preferred Reporting Items for Systematic Reviews and Meta-Analyses (PRISMA) statement [15].

### 2.1. Search Strategy

Every publication in English that was reviewed for this study was extracted from the PubMed, Scopus, EMBASE, and Web of Science databases restricted to the Medical Subjects Headings Index (MeSH/DeCS) to April 2020. The search was based on different combinations of the following keywords: “essential oil”, “oils, essential,” “essential oils,” “volatile oils,” “inflammation,” “inflammations,” “innate inflammatory response,” “inflammatory response innate,” and “innate inflammatory responses.” Furthermore, we reviewed the references in the selected articles for additional reports not included in the original article search.

### 2.2. Study Selection

The two authors independently extracted and proofread the titles and abstracts of each article. The inclusion criteria were the effects of volatile oils and the active components of traditional Chinese medicine on inflammatory diseases, including in vivo and in vitro models and the possible mechanisms of action. The authors excluded articles based on the following criteria: review articles, meta-analyses, abstracts, editorials/letters, conference proceedings, case reports, studies in humans, and articles published more than 5 years ago. The differences of opinion between the two authors were independently decided by the third author.

### 2.3. Data Extraction

One author summarized the data from the article, and the other author examined it.  Table 1 summarizes the following information from the in vitro experiments: the source of the essential oil, species, inflammation type, test index, cell line, the proposed mechanism (s) of biochemical effects, and conclusion.  Table 2 summarizes the following information from the in vivo experiments: the source of the essential oil, species, inflammation type, test index, experimental animal species, the proposed mechanism (s) of biochemical results, and conclusion.

### 2.4. Methodological Quality Assessment

Preclinical in vivo studies of bias risk and quality were based on an optimized checklist [15, 31]. This survey mainly involved the randomization of animal feeding and distribution, blind administration, blind results, and other factors used to evaluate the included methodology.

### 2.5. Data Analysis

Due to the heterogeneity of this study, pooled statistics and meta-analysis were not used. The data analysis is presented in narrative form.

## 3. Results and Discussion

### 3.1. Search Results

**Figure 1:**
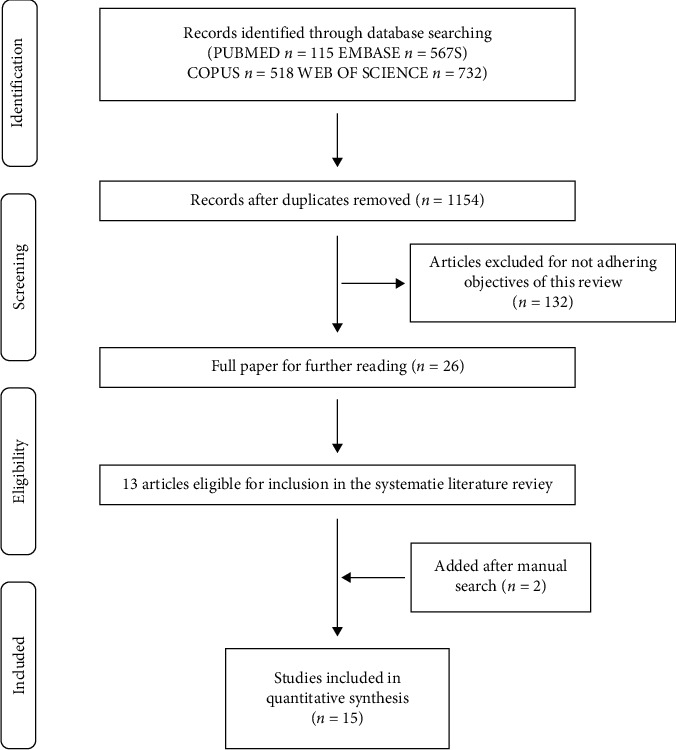
A flowchart of the literature search and selection in this review is described in detail.

Figure 1 presents a flow chart of the search; 1154 articles were identified (PubMed: 115, EMBASE: 567, Scopus: 518, and Web of Science: 732) after duplicate articles had been deleted. After reading the title, we removed articles that were unrelated to essential oils and inflammation and 132 were thus retained. Finally, 15 articles were selected after full-text browsing, among which 12 articles included in vivo experiments, 9 articles included in vitro experiments, and 7 articles included in vivo and in vitro experiments.

### 3.2. Study Characteristics and Description

**Figure 2:**
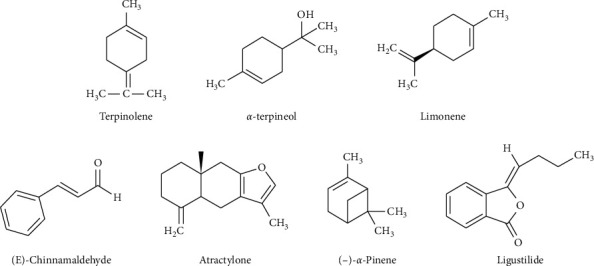
Chemical structure of volatile oil in Chinese traditional medicine.

There were 12 in vivo and 9 in vitro experiments. The influence of essential oil and its components on the inflammatory disease model was investigated in vitro using an inflammatory disease model constructed with a variety of cell lines. Using in vivo experiments, 12 articles studied the effects of traditional Chinese medicine essential oils and their active components on a variety of inflammatory disease models based on mice, rats, or rabbits. The oils showed a significant inhibitory effect on inflammation. Part of the chemical structure of traditional Chinese medicine essential oils is shown in Figure 2. Of the studies examined, 9 articles were from China, 2 were from India, 1 was from Iran, and 1 was from South Korea.

In vitro experiments were performed in human aortic endothelial cells (HAECs), a human keratinocyte cell line (HaCaT), the THP-1 cell line, mouse primary splenocytes and peritoneal macrophages, RAW264.7 cells, and ANA-1 cells. Many in vitro experimental methods were reported in the articles, including immunohistochemical analysis, apoptosis assay, and histological assessment, and proinflammatory cytokines were quantitatively measured by ELISAs, with western blotting and RT-PCR used to detect the expression of various proteins and mRNA, respectively.

In vivo models of inflammation included dextran sulfate sodium (DSS) induced intestinal inflammation, TPA-induced mouse skin inflammation, carrageenan-induced paw oedema, xylene-induced ear oedema model, 2, 4-dinitrofluorobenzene (DNFB)-induced allergic contact dermatitis (ACD), and ischaemic renal rat models. A number of experimental methods were used in the in vivo experiments, including the acetic acid-induced writhing test, oxytocin-induced dysmenorrhea in mice, formalin test, complete Freund's adjuvant-induced overt pain test, carrageenan-induced mechanical hyperalgesia test, evaluation of cytokine levels by ELISAs, and western blot analysis. Through the aforementioned experimental methods, it was further shown that Chinese traditional medicine essential oils have obvious attenuating effects on various kinds of inflammation.

#### 3.2.1. Anti-Inflammatory Effects of Traditional Chinese Medicine Essential Oils In Vitro

An inflammatory response is a symptom of many diseases caused by bacteria or viral infection, physical stimuli, chemical stimuli, and trauma and is a complex biological response to harmful stimuli [32]. Lipopolysaccharide (LPS) is a component of the cell wall of Gram-negative bacteria that causes inflammation by activating MAPK, nuclear factor-kB (NF-kB), and activator protein-1 (AP-1) signalling pathways [18]. In the inflammatory response of cells, COX-2 inhibition exerts a strong anti-inflammatory effect [33]. COX-2 overexpression can also be used as an inflammatory model.

The essential oil from *Gynura procumbens* (GPEO) and its three active components, *a*-pinene [34], 3-carene, and limonene, [35] can inhibit the inflammatory cell infiltration induced caused by noxious stimuli [36]. They have a significant pharmacological effect on the migration of RAW264.7 macrophages induced by LPS and inhibit COX-2 overexpression [37]. Because of its good permeability and anti-inflammatory properties, GPEO is a great treatment for relieving skin redness or treating poisonous insect bites.

The essential oil from *Artemisia argyi* (AAEO) [38] can inhibit the release of inflammatory mediators (NO and PGE2) and the expression of cytokines (IL-6, IFN-*β*, IL-10, and MCP-1). AAEO inhibited the expression of iNOS and COX-2 at the transcription level without affecting its activity [39]. Moreover, AAEO also inhibited the phosphorylation of STAT1 (Tyr701) and STAT3 (Tyr705) and downregulated JAK/STAT signalling and ROS scavenging. ROS production plays a key role in the activation of JAK/STATs in macrophages. According to a previous report, essential oils scavenging ROS have antioxidant activity; therefore, the inhibitory effect of AAEO on JAK/STAT may be due to its antioxidant activity, especially its effect on ROS production [14, 40].

In intestinal inflammation diseases, the levels TNF-*α*, IL-1*β*, IL-6, and IL-12 are significantly higher than those in noninflamed intestines [41]. Intestinal inflammation is often accompanied by mucosal immune system disorders. Researchers found that Maqian fruit essential oil (MQEO) reduced LPS-stimulated expression of TLR4 in THP-1 cells and effectively suppressed the production of IL-1*β* and TNF-*α* in a dose-dependent manner, and in the past, researchers found that curcumin and ellagic acid [42], which are naturally occurring plant phenols, exhibit anti-inflammatory activity by preventing I*κ*B degradation [42]. Linalool is the main component of the essential oil from the blossoms of *Citrus aurantium* L. var. amara Engl (CAVAO) [43]. It can suppress inflammatory symptoms by suppressing NF-*κ*B P65 translocation. It blocks the phosphorylation of IKK and I*κ*B [44, 45]. The anti-inflammatory effect of MQEO may also be related to the TLR4-mediated NF-*κ*B signalling pathway. These two kinds of traditional Chinese medicine essential oils have very good prospects in intestinal inflammatory diseases.

Essential oil extracted from fructus *Alpinia zerumbet* (EOFAZ) [46] changes the expression of ICAM-1 and VCAM-1 and promotes the adhesion of inflammatory cells. Target genes [47] for NF-*κ*B include ICAM-1 and VCAM-1. Atherosclerosis is characterized by chronic systemic inflammation and the formation of large and medium-sized atherosclerotic plaques. The amelioration of inflammation plays an important role in the treatment of this disease [48, 49]. Endothelial cells can promote the adhesion of inflammatory cells by changing the expression of ICAM-1 and VCAM-1 [50], which is believed to play an important role in the occurrence of inflammation and the pathogenesis of atherosclerosis [51]. EOFAZ may serve as a new drug source for the treatment of atherosclerosis.

Mast cells play important roles in allergic inflammation [52]. The essential oil from *Zanthoxylum coreanum* Nakai (ZCO) has been shown to effectively inhibit mast cell degranulation and reduce the level of IL-4, a key inflammatory factor in allergy symptoms [53]. Thirty-seven active ingredients identified from ZCO, mainly *ß*-ocimene and (-)-*α*-pinene, can alleviate allergic inflammation by suppressing the MAPK and NF-*κ*B signalling pathways. ZCO has good transdermal absorbability [54] and will likely be used to treat allergic inflammation in the future.

The skin is the largest sense organ in humans and plays an important role in immune defense [55]. An inadequate immune defense can cause some types of dermatitis. Skin inflammation is closely related to the levels of cytokines and reactive oxygen species [56]. The essential oil from *Curcuma longa* (EOCl) [57] significantly reduced the levels of TNF-*α*, IL-6, and IL-1 in inflammatory models. The main components of EOCl are terpinolene and phellandrene. The essential oil from *Citrus limetta* Risso peels (Clp-EO) is also a candidate for treating skin inflammation [58]. Its main ingredient is limonene, which is not cytotoxic. It can inhibit the levels of TNF-*α*, IL-6, and IL-1*β* in inflammation models and can reduce the levels of ROS under oxidative stress conditions. These two kinds of Chinese herbal essential oils can reduce the levels of various oxidative stress markers. They play antioxidant roles, including a therapeutic role in skin inflammation.

The essential oils extracted from crude *A. macrocephala* (CA) and bran-processed *A. macrocephala* (BA) are CAEOs and BAEOs [59]. The main components of these two essential oils are Atractylone [60]. Atractylone effectively inhibited the level of NO in an allergic rhinitis (AR) model [61]. Atractylone can reduce AR biomarkers and exert a certain anti-inflammatory effect. CAEOs and BAEOs not only exert anti-inflammatory effects but also inhibit gastric cancer, intestinal cancer, and liver cancer cells [62]. These two essential oils have the potential to treat AR. Essential oils can be inhaled into the nasal cavity, which is the best route of administration [63]. They will be the preferred raw materials for the treatment of AR in the future.

*Acorus gramineus* (AG) and *Evodia ruticarpa* (ER) steam-distilled essential oils (SDEOs) are rich in phytochemicals such as total flavonoids, polyphenols, and saponins. The balance between T helper 1 (Th1) lymphocytes and T helper 2 (Th2) lymphocytes is important for human health [64]. These two essential oils can regulate the Th1 and Th2 immune balance and inhibit the TNF-*α*/IL-10 cytokine ratio during the inflammatory response of macrophages, showing a certain anti-inflammatory ability [65].

#### 3.2.2. Anti-Inflammatory Effects of Traditional Chinese Medicine Essential Oils In Vivo

For in vivo tests of essential oils of traditional Chinese medicine with respect to anti-inflammatory diseases, most articles reported the use of chemical reagents to induce inflammation. Essential oils of Chinese medicine have inhibitory effects on inflammation induced by chemical agents. They play inhibitory roles in inflammatory diseases such as skin inflammation and intestinal inflammation, 12-O-tetradecanoylphorbol-13-acetate (TPA) [66] can be used to induce skin inflammation in vivo [67]. After 4 hours of skin exposure to TPA, COX-2 expression in the skin increased significantly, and ear thickness increased significantly. Histological analysis of mouse ear slices can be used to observe the occurrence of inflammation. Skin oedema after successful modelling is obvious, showing many infiltrating neutrophils. This ear oedema model is an ideal in vivo model for screening anti-inflammatory drugs. The essential oils from *Gynura procumbens*, *Citrus limetta* Risso, *Curcuma longa,* and *Artemisia argyi* use the TPA-induced inflammation model in mice, and all four essential oils can reduce the degree of ear swelling in mice [21]. The overproduction of oxidative markers can be regulated by reducing the expression level of COX-2 protein—improved levels of proinflammatory cytokines (TNF-*α*, IL-6, IL-1). Immunohistochemical methods have shown that Chinese medicine essential oil can restore histopathological damage to a great extent. It plays a significant role in inhibiting inflammation.

In the essential oil extracted from *Cinnamomum cassia* Presl, a variety of substances, such as benzenepropanal, (E)-cinnamaldehyde and turmerone, were detected [26]. They can reduce the inflammatory response after different kinds of stimuli. After formalin and CFA were injected into the foot of the mice [68], the mice that took oral oil significantly improved their paw licking behavior. At the same time, the levels of TNF-*α*, NO, IL-1*β,* and PGE 2 in paw oedema tissues induced by carrageenan were reduced in a dose-dependent manner [68]. EO showed an inhibitory effect on acetic acid-induced abdominal contraction. The expression of COX-2 and iNOS in the skin tissues of the feet of mice with essential oils decreased. This shows that this essential oil has a certain anti-inflammatory effect.

Ischemia reperfusion (I/R) injury is a major problem that occurs after a kidney transplant, and it can lead to inflammation [69]. Lavender oil has a certain anti-inflammatory effect on a rat kidney I/R injury model [29]. Oral essential oil can improve renal function by reducing the level of oxidative stress in the pathological cascade caused by renal I/R. Lavender oil can effectively resist renal I/R injury by targeting oxidative stress and apoptosis.

In in vivo models of intestinal inflammation, DSS is generally used to stimulate the intestinal epithelial cells of mice [70], causing mice to produce various symptoms of intestinal inflammation, such as diarrhoea and granulocyte infiltration. Maqian essential oil can reduce MPO and MMP-9 levels in inflammatory tissues, and its anti-inflammatory activity may be related to the NF-*κ*B signalling pathway.

Acetic acid [71] is a common chemical that causes acute colitis. Injection of acetic acid into the colon cavity can cause acute inflammation of colon tissue. TNF-a is closely related to ulcerative colitis. Anise essential oil reduces inflammation by regulating TNF-a levels and inhibiting acetic acid-induced myelin peroxidase activity in tissues [71].

In the LPS-induced in vivo inflammation model, both Angelica essential oil significantly affected the expression of cytokines, inflammatory mediators, and inflammation-related enzymes in the inflammation rat model. This occurs mainly by regulating the expression of the endothelial adhesion molecules ICAM-1 and VCAM-1, thereby preventing the inflammation induced by LPS.

For establishing skin inflammation models, a variety of chemicals can be used to cause skin inflammation, such as xylene-induced ear oedema, carrageenan-induced paw oedema, DNFB-induced [72] allergic contact dermatitis (ACD), DNCB-induced ear swelling, and AD-like skin lesion models. Essential oils can be used to evaluate the anti-inflammatory effects of anti-inflammatory drugs in vivo. Ginger essential oil can protect against xylene-, carrageenan- and DNFB-induced cutaneous inflammation. The essential oil from *Zanthoxylum coreanum* Nakai inhibited DNCB-induced ear swelling and AD-like skin lesions.

### 3.3. Methodological Quality/Risk of Bias

**Figure 3:**
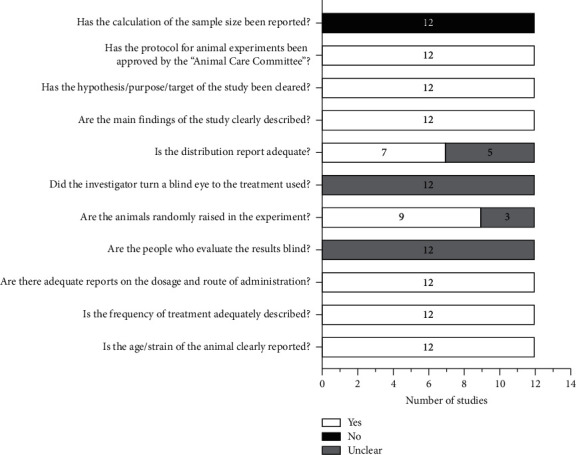
Methodological quality of included in vivo studies.

Figure 3 introduces the methodological features of this review article. The pedigree, frequency of treatment, administration route, and dose of the oils given to experimental animals were clearly described in all the articles. The main objectives and findings of the studies were clearly expressed. However, not all reports reported blind-assessment procedures. Only a few articles reported blind breeding and blind allocation of experimental animals.

**Figure 4:**
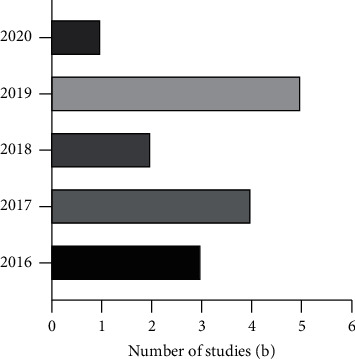
The year of publication of the review article.

**Figure 5:**
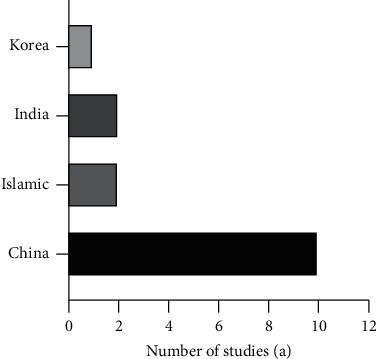
The country of publication of the review article.

Figure 4 and Figure 5 describe the year and country of publication of each article reviewed. Traditional Chinese medicine, as a medicinal material with Chinese herbs, is widely studied not only in China but also in India, South Korea, and Iran. This breadth of study reflects the contribution of traditional Chinese medicine to the development of new drugs around the world. Overall, China has contributed much of value to the world, and traditional Chinese medicine is one of these contributions. Traditional Chinese medicine has been widely used around the world, promoting the development of new drugs based on plant species. As identified by the year of publication, the number of publications has increased gradually in recent years, indicating that the use of traditional Chinese medicine essential oils as anti-inflammatory agents has attracted increasing attention from researchers around the world.

## 4. Conclusion

**Figure 6:**
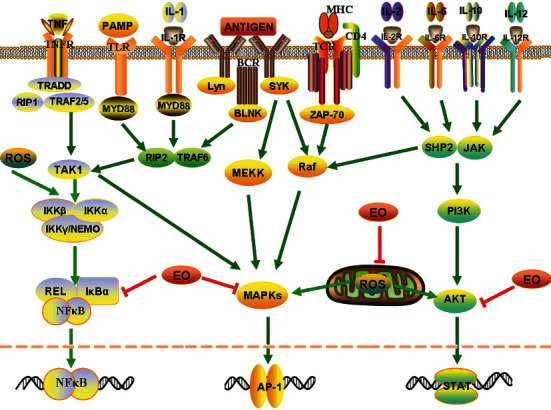
The anti-inflammatory effect of the essential oils of traditional Chinese medicine involves multiple signal pathways.

This review shows that Chinese herbal oils have great potential as naturally extracted compounds in the treatment of inflammatory diseases. The essential oils of traditional Chinese medicine have shown good anti-inflammatory effects in various inflammatory disease models. The essential oils of traditional Chinese medicine can regulate the levels of various cytokines and inhibit multiple signalling pathways that trigger responses to inflammatory diseases, as shown in Figure 6. Most of the articles reviewed in this article were of medium quality, and there are still areas for improvement in some areas. However, overall, the essential oils of traditional Chinese medicine will likely play important roles in the development of new anti-inflammatory drugs in the future.

In 15 articles, we found that essential oils of traditional Chinese medicine have excellent performance in inhibiting signal pathways such as NF-*κ*B, MAPK, and AKT. Numerous antigens will be recognized by the corresponding T cell receptors, B cell receptors, and Toll-like receptors, cytokines such as TNF and IL will be recognized by TNF receptors and a variety of IL receptors, which will trigger the three inflammatory signal pathways mentioned above. In addition, oxidative stress can also trigger inflammation signalling pathways. These three common inflammatory signal pathways are inhibited, expounding the anti-inflammatory mechanism of traditional Chinese medicine essential oils.

## Figures and Tables

**Table 1:** In vitro study of Traditional Chinese medicine essential oils under inflammatory conditions.

Essential oil	Species	Cell types	Inflammation of the species	Tested indicators	Assays biochemical	Molecular	Conclusion	Reference
Maqian fruits essential oil	Rutaceous	THP-1 cell line	LPS-induced	TNF-*α*, IL-1*β*, IKK, I*κ*B	ELISA	Western blot	This essential oil inhibits inflammation by lowering levels of inflammatory factors (TNF-*α* and IL-1*β*) and preventing phosphorylation of IKK and I*κ*B	[16]
Essential oil from waste leaves of Curcuma longa L.	Zingiberaceae	Human keratinocyte cell line (HaCaT)	LPS and TPA-induced	TNF-*α*, IL-6, IL-1*β*	—	RT-PCR	Under the premise of no toxicity, this essential oil can inhibit the level of inflammatory factors (TNF-*α*, IL-6, IL-1*β*) and further inhibit the inflammatory response	[17]
Essential oil from Fructus Alpinia zerumbet	Zingiberaceae	Human aortic endothelial cells	LPS-induced	ICAM-1, VCAM-1	-	PCR, western blot	This essential oil prevented LPS-induced HAEC activation and inflammation.	[18]
Essential oil from zanthoxylum coreanum nakai	Rutaceae	RAW264.7 cells	LPS-induced	TNF-*α*, IL-6,	ELISA	—	This essential oil inhibits inflammation by regulating the NF-*κ*B and MAPKs signalling pathways	[19]
Essential oil from acorus gramineusand and euodia ruticarpa	Acorus and tetradium	Mouse primary splenocytes and peritoneal macrophages	LPS-induced	Th1, IL-2, Th2 IL-5, TNF-*α*, IL-10	ELISA	—	These two essential oils have the potential to regulate Th1/Th2 balance of spleen cells and suppress macrophage inflammation	[20]
Essential oils from gynura procumbens	Compositae	RAW264.7 cells	LPS-induced	COX-2	ELISA	—	This essential oil can suppress nociceptive inflammation by regulating COX-2 levels	[21]
Essential oil from artemisia argyi	Artemisia	RAW264.7 cells	LPS-induced	NO, PGE2, ROS, TNF-*α*, IL-6, IFN-*β* and MCP-1	—	PCR, western blot	This essential oil inhibits inflammation through down-regulation of the JAK/STATs signalling	[22]
Essential oil from atractylodes macrocephala	Compositae	ANA-1 cells.	LPS-induced	NO	—	—	This essential oil reduces NO levels after stimulation and suppresses inflammation	[23]
Essential oil from citrus limetta;	Citrus L.	RAW264.7 cells	LPS-induced	TNF-*α*, IL-6, IL-1*β*	ELISA	—	This essential oil can inhibit inflammation by inhibiting the expression of a variety of cytokines	[24]
Essential oil from citrus aurantium L. var. amara	Citrus aurantium L. var. amara engl	RAW264.7 cells	LPS-induced	iNOS, IL-6, TNF-*α*, IL-1*β* COX-2.	—	Western blot RT-PCR	This essential oil inhibits inflammation by regulating the NF-*κ*B and MAPKs signalling pathway	[25]

**Table 2:** In vivo study of Volatile oil from Traditional Chinese medicine under inflammatory conditions.

Essential oil *Substance*	Species	Animal	Inflammation of the species	Tested indicators	Assays Biochemical	Molecular	Conclusion	Reference
Maqian fruits essential oil	Rutaceous	Mice	DSS- induced intestinal inflammation	IL-1*β*, IL-6, IL-12, p35, TNF-*α*	—-	RT-PCR	This essential oil inhibits DSS-induced intestinal inflammation by regulating the TLR4-mediated NF-*κ*B-related signalling pathway.	[16]
Essential oil from waste leaves of Curcuma longa L.	Zingiberaceae	Mice	TPA-induced skin inflammation	TNF-*α*, IL-6,, IL-1*β*	ELISA	—	Topical application of this essential oil can reduce the ear thickness of ear swelling and ameliorating the level of TNF-*α*, IL-6, IL-1*β* cytokines in the ear swelling tissue.	[17]
Cinnamomum Cassia essential oil	Lauraceae	Mice	Paw edema induced by carrageenan	TNF-*α*, IL-1*β*	ELISA	Western blot	This essential oil can improve the swelling of mice feet by regulating the expression of cytokines ““ (TNF-*α* and IL-1*β*), NO, and PGE2.	[26]
Ginger essential oil	Zingiberaceae	Mice	Xylene-induced ear edema model, carrageenan-induced paw edema model and DNFB-induced allergic contact dermatitis (ACD) model	TLR-2, TLR-4, TNF-*α*, IFN-*γ*, IL-1*β*, IL-8, IL-4	—	RT-PCR	This essential oil can reduce the expression of TLR-2, TLR-4, TNF-*α*, IFN-*γ*, IL-1*β*, IL-8and increase the expression of IL-4 to fight skin inflammation.	[27]
Angelica sinensis essential oil	Angelica	Mice	LPS-stimulated	TNF-a, IL-10, IL-6, IL-1*β*	ELISA	—	This essential oil can regulate the cytokines, mediators and enzymes in the inflammatory model and play a good anti-inflammatory role.	[28]
Essential oils from gynura procumbens	Compositae	Mice	Xylene-induced ear oedema and hind paw model.formalin-injected mice	COX-2	—	—	This essential oil can play an anti-inflammatory role in the ear edema model, the plantar edema test, and the formalin-injected mice inflammation model	[21]
Essential oil of artemisia argyi	Artemisia	Mice	TPA-induced mouse ear edema	COX-2	—	Western blot	This essential oil reduces TPA-induced ear edema by lowering COX-2 protein levels.	[22]
Essential oil from citrus limetta;	Citrus	Rabbit, mice	TPA-induced mouse ear inflammation	TNF-*α*, IL-6, IL-1*β*	ELISA	—	This oil is nonirritating to the skin of rabbits and inhibits TPA-induced ear inflammation by reducing cytokine levels (TNF-, IL-6, and IL-1).	[24]
Essential oil fromLavandula angustifolia	Labiatae	Rats	A rat model of renal ischemia	TNF*α*, IL1*β*, IL10	ELISA	—	This essential oil can reduce the level of TNF*α*, IL1*β*,, increase the level of IL10. Furthermore, it can restore the activity of antioxidant enzymes, reduce acute inflammation, and reduce rejection in kidney transplant patients.	[29]
Essential oil from foeniculum vulgare	Foeniculum	Rats	Acetic acid-induced rat colitis	TNF-a	—	Western blot	This essential oil has an anti-inflammatory effect on colitis rats, possibly by regulating the NF-kB pathway	[30]
Essential oil from fructus alpinia zerumbet	Zingiberaceae	Mice	LPS-stimulated	ICAM-1, VCAM-1, NF-*κ*B, p65, (Phospho-p65)	—	Western blot	This essential oil alleviates lPS-induced endothelial injury by regulating endothelial adhesion molecules and regulating NF-kB signalling.	[18]
Essential oil from fruits of zanthoxylum coreanum nakai	Zanthoxylum	Mice	DNCB-induced atopic dermatitis model	NF-*κ*B, p65, phosphorylated JNK, ERK, p38	—	Western blot	This essential oil reduces ear swelling and skin damage in mice through inhibition of NF-kB activity and MAPKs phosphorylation.	[19]
